# Electrochemotherapy with cisplatin enhances local control after surgical ablation of fibrosarcoma in cats: an approach to improve the therapeutic index of highly toxic chemotherapy drugs

**DOI:** 10.1186/1479-5876-9-152

**Published:** 2011-09-14

**Authors:** Enrico P Spugnini, Sylvie M Renaud, Sabrina Buglioni, Francesca Carocci, Emanuele Dragonetti, Raffaele Murace, Pierluigi Cardelli, Bruno Vincenzi, Alfonso Baldi, Gennaro Citro

**Affiliations:** 1 SAFU Department, Regina Elena Cancer Institute, Rome, Italy; 2 Ambulatorio Veterinario Renaud, Rome, Italy; 3 Ambulatorio Veterinario "Le Accademie", Rome, Italy; 4 Futura-Onlus, Rome, Italy; 5 Tor Vergata University, Rome, Italy; 6 University Campus Biomedico, Rome, Italy; 7 Department of Biochemistry, Second University of Naples, Naples, Italy

## Abstract

**Background:**

Cancer is one of the most difficult current health challenges, being responsible for millions of deaths yearly. Systemic chemotherapy is the most common therapeutic approach, and the prevailing orientation calls for the administration of the maximum tolerated dose; however, considerable limitations exist including toxicities to healthy tissues and low achievable drug concentrations at tumor sites. Electrochemotherapy (ECT) is a tumor treatment that combines the systemic or local delivery of anticancer drugs with the application of permeabilizing electric pulses. In this article we evaluate the capability of ECT to allow the use of cisplatin despite its high toxicity in a spontaneous feline model of soft tissue sarcoma.

**Methods:**

A cohort of sixty-four cats with incompletely excised sarcomas were treated with cisplatin-based adjuvant ECT and monitored for side effects. Their response was compared to that of fourteen cats treated with surgery alone.

**Results:**

The toxicities were minimal and mostly treated symptomatically. ECT resulted in increased local control (median not reached at the time of writing) with a mean time to recurrence of 666 days versus 180 of controls.

**Conclusions:**

We conclude that ECT is a safe and efficacious therapy for solid tumors; its use may be considered as part of strategies for the reintroduction of drugs with a narrow therapeutic index in the clinical protocols.

## Introduction

Cancer is among the major causes of death in the human population, being responsible for millions of deaths each year [1,2]. Systemic chemotherapy is the most commonly used therapeutic strategy, although considerable limitations exist since conventional chemotherapy often involves pulsatile administration schedules using maximum tolerated doses (MTDs) of cytotoxic drugs. The long break periods between therapies not only allow recovery from various toxicities, especially myelosuppression, but also provide an opportunity, unfortunately, for the drug-treated tumors to recover as well [3-8]. The therapeutic index (also known as therapeutic ratio), is a comparison of the amount of a therapeutic agent that causes the therapeutic effect to the amount that causes death (in animal studies) or toxicity (in human studies). Some drugs have such a narrow therapeutic index to be associated with significant systemic side effects, including gastro-intestinal toxicity, cardiac toxicity and bone marrow depletion, that could result, among other complications, in hemorrhage and sepsis [9-11]. Furthermore, systemic chemotherapy is often not efficient in delivering drugs to target sites at therapeutic concentrations, and maintaining adequate drug levels within tumors is a challenge [2-14]. Chemotherapeutic delivery to solid tumors systemically involves several limiting factors, including the role of the i) drug transport along the blood circulatory system to tissues (including also the issue of plasma binding proteins), ii) interstitial space, iii) drug removal by capillaries and, last but not least, iv) tissue structure and composition with respect to the drug distribution [1,12-14]. As a result, only a fraction of the administered dose reaches tumor cells, which dramatically hinders tumor targeting, prevents effective therapy and increases toxicity to healthy tissue [1,12-14]. Considering that more than 85% of human cancers are solid tumors [8], several strategies have been adopted to overcome these problems, including intra-arterial chemotherapy [15], chemotherapy impregnated implants [16-18] and polymeric drug delivery systems [8]. Despite the continuous investigation of alternate routes for improved systemic chemotherapeutic delivery there has been minimum therapeutic gain [8,15-18], therefore localized delivery has gained increased attention in cancer therapy. This strategy aims at maintaining low systemic drug levels while ensuring therapeutic levels at target sites, thus aiming to deliver a much more efficient drug therapy [8,14]. Selective drug retention at target sites, with consequent decreased systemic drug exposure, is an important issue, since two therapeutic goals are minimizing toxicity and maximizing efficacy [19]. This selectiveness can be accomplished through electrochemotherapy (ECT) a cancer treatment that involves the application of permeabilizig electric pulses having appropriate waveforms coupled with the local or systemic administration of chemotherapy agents [20,21]. Over the past years, several ECT studies have been conducted on companion animals affected by advanced spontaneous tumors, obtaining high response rates, while surgery alone, especially for rapidly growing tumors such as feline sarcomas results in control times ranging from 60 to 270 days [22-25]. Cisplatin is a chemotherapy agent widely adopted in veterinary oncology that cannot be administered systemically to cats since it induces severe pulmonary toxicoses including dyspnea, hydrothorax, pulmonary edema, mediastinal edema and death [26,27]. Aim of this investigation is the evaluation of toxicoses and efficacy of local cisplatin as agent for adjuvant ECT in a spontaneous feline sarcoma model.

## Methods

The Regina Elena Cancer Institute Ethical Committee approved this study that was performed according to the Italian Law (116/92). Seventy-eight privately owned cats with histopathologically confirmed incompletely excised or recurring soft tissue sarcoma (STS) were entered in the modified phase II study. Evidence of recurrence was demonstrated upon the diagnostic histopathology analysis of bioptic specimens excised by the referring veterinarians. Previous informed consent was obtained from the owners. In order to be enrolled in the study, patients had to fulfill the following criteria:

1. Accessibility of the neoplasm location.

2. Absence of distant metastases.

3. Compliance of the owner for follow-up rechecks.

4. Absence of other life-threatening diseases.

5. Overall performance status, assessed according to the modified Karnowsky system, had to be less than 3 [19,23].

Staging process included a thorough anamnesis, histopathologic assessment of surgical margins, physical examination, complete blood cell count (CBC), serum biochemistry profile, urinalysis thoracic radiographs (three projections), and abdominal ultrasonography. In order to confirm the diagnoses, histological examination of the biopsies was performed following standard protocols, using Hematoxylin/Eosin and Hematoxylin/Van Gieson stainings. Patients receiving adjuvant ECT were matched with a cohort of cats treated with surgery alone, having the owners declined to pursue other treatments than surgery.

### Treatment

Patients received two rounds of ECT 1 week apart at a voltage of 1,300 V/cm delivered through caliper electrodes, beginning 1 week after tumor excision. The surgical suture and 3 cm of apparently normal tissue (up to 1.5 cm depth) were locally injected with cisplatin at a concentration of 0.5 mg/ml. All the ECTs were given after sedation with medetodimine and ketamine following the manufacturers' instruction followed by administration of propofol. Five minutes after the infiltration of the anticancer agent, trains of eight biphasic electric pulses lasting 50 + 50 ms each, with a frequency of 1 Hz, and with 1-ms interpulse intervals (total treatment time: 7.1 ms/cm^2 ^of treated area), were generated by a Chemipulse III portable electroporator (EU patent application number 2221086) and delivered by means of modified caliper electrodes with 1 cm distance between the plates.

### Follow-up evaluations

A CBC, serum biochemistry profile and urinalysis were performed prior and 1 week after each dose of cisplatin. A monthly recheck was scheduled after the completion of the treatment. Thoracic radiographs were taken on a 3 months base until a 1 year follow-up was reached. Recurrence was proved by cytopathology or histopathology.

### Endpoints

During the ECT sessions the patients were checked using cardiac monitor and pulse oxymeter. The primary endpoint was the evaluation of cisplatin-related toxicoses (cutaneous, respiratory, gastrointestinal, hematological or renal); the secondary endpoint was the time of tumor recurrence. However, at the time of tumor recurrence, a second course of surgery and ECT was offered to the owner considering its high success rate in selected patients [23,25].

### Statistical analysis

A univariate survival analysis for each prognostic variable on overall survival was estimated according to the Kaplan-Meier method [28]. The terminal event was tumor recurrence. The mean time to recurrence is described as the median time and the mean time (adding in the last case the range). The statistical significance of the differences in control time among the prognostic groups was evaluated by the log-rank test [29]. P values < 0.05 was regarded as statistical significant in two-tailed tests. SPSS software (version 11.5, SPSS, Chicago) was used for the statistical analysis.

## Results

Sixty-four cats entered the electrochemotherapy study over a 5 years period and their response has been matched with that of 14 cats treated with surgery alone.

### Hematological, gastrointestinal and renal toxicity

Hematological toxicity was not evidenced among the 64 patients of the ECT study. Three patients (4.5%) developed gastrointestinal symptoms, mostly consisting of anorexia lasting 3-5 days, responsive to anti-emetic therapy with metoclopramide. One patient, a seven-year-old cat, died of renal failure, probably unrelated to the treatment as suggested by the current literature [23].

*Muscular side effects *All the patients experienced transient muscular contractions at the time of the treatment, which were more pronounced in those cats whose tumor was located nearby nerve roots. These contractions did not result in pain or discomfort for the patients when they recovered from the sedation and did not require symptomatic therapy.

*Cardiorespiratory side effects *Neither cardiac disturbances related to the electrical stimulation nor pulmonary distresses related to the administration of cisplatin were evidenced during the study.

*Cutaneous side effects *Despite the use of electroconductive gel, seven patients had small electrode-induced burns which exited in 1 cm long, 1 mm wide discolored scars. These scars tended to disappear within 2-3 weeks as previously reported [23,25].

*Soft tissue side effects *Three cats had a local inflammation involving the deep subcutaneous connective tissues, which led to compulsive scratching. This phenomenon, in one patient, led to wound dehiscence that needed re-suturing. In the other two cats the inflammation was successfully managed through the administration of the non steroidal anti-inflammatory drug meloxicam for a period of one week.

### Treatment efficacy

**Figure 1:**
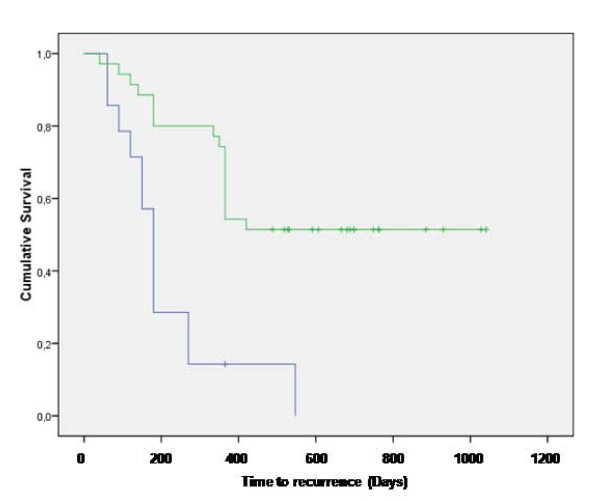
**Treatment outcome: Kaplan-Meier time to recurrence curve for cats treated with surgery (blue line) and cats treated with the combination of surgery and ECT (green line)**.

Out of the 14 cats that underwent surgery only, one patient is still in remission (in excess of one year) at the time of writing, while the remaining 13 cats had recurrence of the tumor at different times. Median time to recurrence was 180 days (Figure 1); mean time to recurrence was 213 days (range 131-294 days). Due to the aggressive nature of the tumors, the owners of these cats declined further treatments at the time of local recurrence.

Out of the 64 cats enrolled in the ECT study, eight died of the following unrelated pathologies: four died of causes related to advanced age, one died of renal failure, one died of FeLV infection, one died of metastatic carcinoma and one died of metastatic fibrosarcoma, and therefore were censored in the statistical analysis. Finally, 19 (29.7%) cats had tumor recurrence at different times. Four of the recurring tumors were amenable to another course of surgery and adjuvant ECT experiencing control times that were of 300 days for one patient (at that time the tumor recurred and the owner declined further therapies), and in excess of 120, 300 and 565 for the remaining three. Median time to recurrence for the ECT cohort was not reached at the time of article writing, while mean time to recurrence was 666 days (range 535-797 days). Figure 1 shows the Kaplan-Meier curve for both cohorts participating the study. The only statistical significant parameter was the ECT treatment (P = 0.0002), on the contrary the following statistical factors failed to reach the statistical significance: gender, age, site of primary tumor and dimension (measures as three-dimensional volume).

## Discussion

Cancer is one of the most difficult health challenges of our time. In addition to the currently available conventional therapeutic modalities such as chemotherapy, radiotherapy and surgery, there is an urgent need for more effective and less toxic therapies for solid malignancies. Chemotherapy alone shows high toxicity and a low survival rate [6,7]. In some cases, malignant cells develop resistance to a particular drug and to combat this, a variety of approaches like intra-arterial therapy, induction chemotherapy, immunotherapy, and photodynamic therapy have been employed [8]. Techniques like intra-arterial and induction chemotherapy have showed some improvement in survival rate. Immununotherapy is in the experimental stages, while photodynamic therapy is being clinically applied, but side effects prevented its diffusion. Various treatment strategies have been explored to improve upon current systemic chemotherapy methods. Localized pulse mediated delivery of chemotherapeutics for solid tumors is one approach that has been explored in recent years due to the customizability of the therapy [20,24]. However, some concerns still need to be addressed. One challenge of localized electrochemotherapy is the lack of control in drug distribution when treating solid tumors. Intravenously delivered chemotherapeutics must overcome barriers including the vasculature wall. Moreover, hypoxic tumor regions distant from vasculature may not be exposed to adequate amounts of drug, thus developing multi-drug resistance. On the other hand, drug penetration following intralesional administration is hindered by the extravascular compartment of tumor tissue, which limits drug diffusion [19,30]. In this study, to overcome the above limitations, ECT has been used in an adjuvant fashion, after tumor resection, thus limiting its purpose to the eradication of the surviving clonogenic cells and the prevention of tumor recurrence. More importantly, the rapid shifting of cisplatin from the interstitial space to within the tissue, prevented potentially fatal toxicities in this feline model of solid tumor. Furthermore, the increased susceptibility of tumor cells to the permeabilizing pulses, compared to that of normal tissues, allows a semi-selective drug delivery [19-25], therefore supporting tumor control while side effects are minimized. The results in terms of local control suggest a therapeutic gain when ECT is added to the surgical protocol and compare favorably with the results obtained by our group in a previous study of ECT in cats with soft tissue sarcomas [23]. In that investigation bleomycin has been used since it is the first choice drug for ECT. Cisplatin is a second choice drug for ECT and its systemic administration in cats is not possible due to fatal pulmonary edema [26,27]. Indeed one of the aims of our study was to evaluate the reintroduction of highly toxic compounds through electroporation in the anticancer protocols. The simple strategy of using electroporation allowed us to greatly increase the therapeutic index of cisplatin in cats, which are very susceptible to this drug. It is conceivable that the increased adoption of this strategy by other investigators will result in the "rescue" of other drugs that otherwise would not be clinically usable, due to excessive systemic toxicity. Also, it is noticeable that only one ECT patient experienced metastatic spread of the primary tumor. The low frequency of this event (1/64, i.e., ~1.6%) compares favorably with what observed in our previous investigation of cats with sarcomas, and more importantly, with the much higher metastatic incidence reported in the literature (depending on the studies it ranges from 10% to 25%) [23].

## Conclusion

ECT is an effective strategy to improve the chemotherapy efficacy and the therapeutic index of chemotherapy drugs in the treatment of solid neoplasms. Further studies with a greater number of patients and, possibly, involving multiple institutions should be performed in order to better elucidate the real clinical impact of ECT on STS treatment in feline patients, also in view of a possible translation to humans [24].

## Competing interests

EPS and AB are among the applicants of the patent for the Chemipulse III portable electroporator (EU patent application number 2221086).

## Authors' contributions

EPS, AB and GC conceived the study and participated in its design and cohordination and wrote the article; SMR, SB and FC helped with the clinical management of the patients, ED, RM, PC and BV participated in the design of the study and performed the statistical analysis. All the authors read and approved the final manuscript.
